# Lignin–Polyethylene Oxide Interlocked Phase Change Materials with Enhanced Thermal Stability and Form Retention for Efficient Heat Management

**DOI:** 10.3390/polym17010044

**Published:** 2024-12-28

**Authors:** Junsang Park, Pranto Karua, Songtao Tang, Ngoc A. Nguyen, Lili Cai

**Affiliations:** 1 Department of Mechanical Science and Engineering, The Grainger College of Engineering, University of Illinois Urbana-Champaign, Urbana, IL 61801, USA; junsang5@illinois.edu (J.P.); pkarua2@illinois.edu (P.K.); songtat@illinois.edu (S.T.); 2 Illinois Applied Research Institute, The Grainger College of Engineering, University of Illinois Urbana-Champaign, Champaign, IL 61801, USA; 3 Materials Research Laboratory, The Grainger College of Engineering, University of Illinois Urbana-Champaign, Urbana, IL 61801, USA

**Keywords:** phase change materials, lignin, polyethylene oxide, interlocked structures, battery thermal management system

## Abstract

The rapid advancement of high-performance technologies, such as electric vehicle (EV) batteries; data centers; and AI systems, has underscored the critical need for effective thermal management solutions. Conventional phase change materials (PCMs) often face challenges, like phase leakage, dimensional instability, and environmental concerns, limiting their effectiveness in high-stress applications. This study introduces a novel PCM composed of polyethylene oxide (PEO) and lignin, developed to overcome the existing limitations while improving overall thermal management performance and promoting material sustainability. By chemically crosslinking lignin with aliphatic polymer chains compatible with PEO during co-reactive melt processing, we created an interlocked structure that combines high heat capacity with exceptional structural stability. This structure allows the PCM to retain its form and resist phase transitions even under elevated temperatures, up to 115 °C, far above the melting point of PEO, effectively mitigating leakage issues common in conventional PCMs. Comprehensive thermal characterization and dynamic performance testing demonstrate that the lignin-modified PEO composites effectively absorb and dissipate heat, maintaining dimensional stability and resilience under repeated thermal cycling. These findings position these composites as sustainable, reworkable, and efficient alternatives for advanced thermal management applications, particularly in battery thermal management systems (BTMSs), where stability, durability, and performance are critical.

## 1. Introduction

As technology accelerates, high-performance systems like electric vehicle (EV) batteries, artificial intelligence (AI) infrastructures, and data centers are generating unprecedented levels of heat, creating critical challenges for performance, safety, and operational efficiency [1,2,3]. In EV batteries, for example, thermal imbalances can lead to hotspots, decreasing battery lifespan, reducing capacity, and even posing fire risks [4]. Similarly, in data centers, ineffective heat management can result in overheating, increasing the likelihood of system failures, downtime, and energy inefficiencies [5]. With such heat-sensitive operations becoming foundational in modern technology, reliable and efficient thermal management is essential to maintaining stability, extending system lifespans, and ensuring the safety of these advanced applications [6].

Conventional cooling techniques, including air and liquid cooling, heat pipes, and evaporative cooling, have been widely used to address these thermal challenges [7,8]. However, these methods often require complex machinery or energy inputs, limiting their effectiveness in compact or noise-sensitive environments, such as EV battery packs or densely packed data centers [9,10]. PCMs have emerged as an alternative solution due to their unique ability to absorb and release large amounts of heat during phase transitions without additional energy input. This characteristic allows PCMs to deliver passive cooling, which is energy-efficient and silent, offering an advantage over conventional cooling methods for a range of applications where design flexibility and noise reduction are critical considerations [11,12,13].

Despite these benefits, commonly used organic PCMs, such as paraffin wax, come with significant limitations [14]. Conventional PCMs tend to undergo a full phase transition to a liquid state at elevated temperatures, commonly above their melting point, which can result in leakage, volume expansion, and form instability [15,16]. Furthermore, organic-based PCMs, such as paraffin wax, raise environmental and safety concerns due to their petrochemical origins and flammability. While recent advancements, such as the development of leakage-proof and ultraflexible polymer-based phase change composites via chemical cross-linking for wearable thermal management (Jing et al.) [15], have demonstrated significant improvements in flexibility and leakage resistance, these approaches primarily address applications in wearable electronics. Our study focuses on extending these advancements to high-stress environments like battery thermal management systems (BTMSs), where structural stability and high thermal performance are crucial under repeated thermal cycling. Additionally, organic PCMs have petrochemical origins and are often flammable, raising environmental and safety concerns, particularly in high-temperature applications [17,18]. These drawbacks limit the long-term reliability and sustainability of the PCMs, underscoring the need for more stable, form-retentive, and environmentally friendly alternatives capable of enduring repeated thermal cycling and fluctuating thermal loads.

This study introduces a novel phase change material composed of PEO and lignin, designed to address those limitations while improving thermal management capabilities. PEO is known for its high heat capacity and moderate thermal conductivity, making it effective for heat absorption and retention [19,20]. However, PEO alone may undergo excessive softening and phase transitions at high temperatures, reducing its practical use as a PCM [21]. Lignin is a natural polymer and a byproduct of the paper and biorefinery industries. It contains rich functional groups and aromatic structural units, offering high molecular rigidity, flame resistance, and environmental sustainability [22,23]. Extensive research on composites of PEO with lignin and its derivatives has uncovered promising potential as thermal management materials or PCMs. Recently, we developed an interlocked lignin–PEO structure using co-reactive melt processing, where the epoxy chain ends of a flexible, hydrogen bond-donating aliphatic structure were crosslinked with functional groups in lignin, simultaneously blended with ultra-high molecular weight PEO [24]. This synthesis yielded materials with significantly enhanced stiffness and multiple functional properties through robust molecular interactions. The morphological properties, crystal structure, and crystallization behavior of the materials are indeed closely related to their rheological and thermal characteristics, which have been thoroughly demonstrated in our previous studies [24,25]. In this paper, we focus on demonstrating the proof of concept for the materials’ capabilities in thermal management system applications. Building on these unique features, we propose employing this interlocked lignin–PEO structure for manufacturing advanced PCMs tailored for thermal management. Leveraging this synthesis technique, we designed and evaluated a series of lignin-modified PEO composites, demonstrating their efficacy in thermal management applications as PCMs.

When combined with PEO, lignin provides a good supporting structure, and stabilizing matrix, enhancing the PCM’s dimensional stability and preventing material leakage, even under applied stress. This integration results in lignin-modified PEO composites with superior heat capacity, structural integrity, and resistance to melting, setting them apart from conventional PCMs. The enhanced stability of this composite is achieved through a chemically interlocked structure formed during synthesis, where crosslinking between lignin and inter- and intra-molecular interactions through hydrogen bonds, pi-pi stacking, and entanglements within lignin and PEO create a robust three-dimensional dynamic network that can withstand high temperatures with enhanced form stability while still retaining good melt processability [24]. The interlocked structure of lignin-modified PEO composites prevents the solid–liquid phase transition and maintains dimensional stability across multiple thermal cycles [26]. This unique design not only prevents the leakage and degradation issues seen in conventional PCMs but also ensures that the materials can be used after cooling, a key advantage for long-term applications requiring maintenance and reusability without structural compromise.

This paper presents a comprehensive analysis of the lignin-modified PEO PCM’s thermal properties and resilience across various conditions. Through a series of experiments, we evaluate their resistance to melting, thermal management performance at high temperatures, and structural stability over repeated thermal cycles. By examining how the lignin-modified PEO composites address the limitations of conventional PCMs, this study positions the composite as a promising alternative for advanced thermal management systems in high-demand applications, such as BTMS, where stable thermal management performance, safety, and environmental compatibility are essential.

## 2. Experimental Methodology

### 2.1. Materials

PEO with an approximate molecular weight of 5,000,000 g/mol, was obtained from Scientific Polymer Products, Inc. (Ontario, New York, NY, USA), and served as the base polymer due to its high heat capacity and thermal conductivity. Kraft lignin, sourced from Sigma-Aldrich (Saint Louis, MI, USA), was chosen for its natural rigidity and flame-retardant properties, enhancing the stability of the composite under thermal cycling conditions.

To promote crosslinking and structural interlocking, trimethylolpropane triglycidyl ether (TTE) and triphenylphosphine (TPP), both supplied by Sigma-Aldrich (Saint Louis, MI, USA), were employed as a crosslinking agent and catalyst, respectively. All chemicals were used as received to ensure reproducibility and reliability in subsequent experiments. Chemical crosslinks and macromolecular interactions within these components resulted in a composite with enhanced dimensional stability and resistance to melt flow and leakage.

### 2.2. Configuration of BTMS and PCM Structures

**Figure 1:**
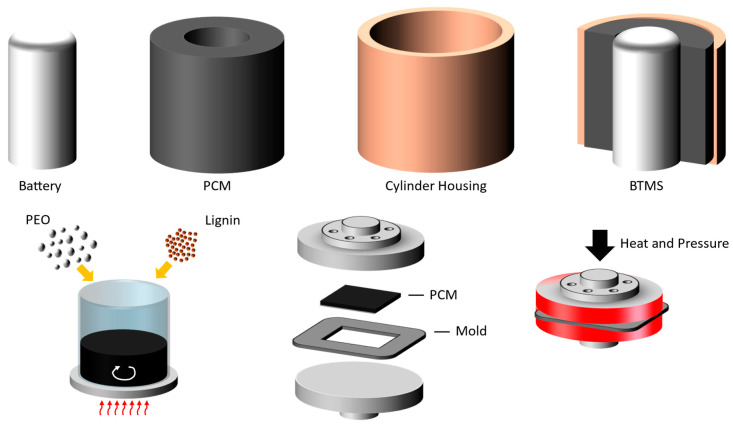
Schematic of the fabrication process and configuration of the battery thermal management system (BTMS) using the lignin-modified PEO-based phase change material (PCM). Polyethylene oxide (PEO) and lignin are combined in a controlled melt-mixing process to form a composite PCM, which is subsequently molded into shape and integrated with the heater and cylindrical housing.

Figure 1 illustrates the configuration of the system. The prepared PCM was integrated into the battery thermal management system (BTMS). In this work, a cylindrical type of electrical heater was used to simulate the battery. The heater has a diameter and height of 15.88 mm and 100 mm, respectively.

For the PCM system, the electrical heater was wrapped by the PCM, and they were contained within a cylindrical housing made of PLA, which had a height of 110 mm, a thickness of 3 mm, and an inner diameter of 33 mm (see Figure 1). The PCMs used in this study were lignin-modified PEO composites. PEO was used as the base polymer, while lignin served as the additive to enhance the thermal and environmental properties of the PCMs. The primary materials used in the synthesis of the lignin-modified PEO composite-based PCM were kraft lignin, trimethylolpropane triglycidyl ether (TTE), and triphenylphosphine (TPP), all obtained from Sigma-Aldrich (Saint Louis, MI, USA). Polyethylene oxide (PEO, Mw ~5,000,000 g/mol) was sourced from Scientific Polymer Products Inc (Ontario, New York, NY, USA). All materials were used as received without further purification.

The PCM was synthesized from PEO and kraft lignin and utilized for the BTMS. The resulting PCM composite was added into a custom-designed mold and pressed at 190 °C to create flat PCM sheets of uniform thickness, ensure structural uniformity, and eliminate voids within the material. The details of PCM synthesis and compression molding are presented in the following sections. The final PCM sheets were trimmed to fit the cylindrical housing before assembly into the BTMS. Figure 1 illustrates the detailed structure of the BTMS, including a model battery (electrical heater), a PCM sheet wrapping around the battery, and a cylinder housing. This BTMS will be used to test the thermal management characteristics of the materials in this study.

### 2.3. PCM Synthesis

The synthesis of the lignin-modified PEO composites was conducted through a co-reactive melt processing technique using a Brabender mixer following our previous method [24]. This process was designed to create an interlocked structure that enhances the thermal properties of the PCMs by combining PEO with crosslinked lignin. Initially, the PEO was loaded into a pre-heated mixing chamber at 180 °C and mixed at 15 rpm for 2 min to ensure uniform melting. Following this, a mixture of kraft lignin and TTE (crosslinker) in a weight ratio of 5:1, along with TTP (catalyst, 1 wt.% of the total lignin and crosslinker mixture), was introduced into the chamber. The combined materials were then mixed at 50 rpm for 10 min to achieve uniform dispersion and crosslinking. The cross-linking process played a crucial role in forming a stable, interlocked structure between the PEO and lignin.

### 2.4. Sample Preparation

Three different formulations were synthesized to investigate the effect of PEO concentration on the PCM’s performance: 40 wt.% PEO, 64 wt.% PEO, and 88 wt.% PEO. These samples were labeled as lignin-modified PEO40, lignin-modified PEO64, and lignin-modified PEO88, respectively. A neat PEO sample (pure PEO without lignin modification) was used as a control for comparison. After synthesis, the resulting materials were compression molded at 190 °C under a pressure of 5 metric tons for 10 min to form solid PCM samples for further characterization. This compression molding process ensures the removal of any voids and enhances the uniformity of the final product, ensuring reproducibility in terms of thermal and mechanical properties [27,28].

### 2.5. Thermal, Rheological, and Chemical Characterization

The thermal properties of the materials were characterized using a differential scanning calorimeter (DSC-Q200, TA Instruments, New Castle, DE, USA) [29]. The testing was conducted within the temperature range of −80 to 150 °C at a rate of 10 °C/min. The melting temperature, latent heat, and specific heat capacity were measured for each sample during the second heating cycle to evaluate the thermal performance. The melt rheology of the samples was investigated using a DHR 30 rheometer (TA Instruments, New Castle, DE, USA). All rheological properties of the materials were characterized in an oscillatory shear mode using 8 mm parallel plates in the linear viscoelastic regions. Oscillatory frequency sweeps of the samples were conducted at two reference temperatures, 80 °C and 150 °C, which are above the samples’ melting points. This step was essential to evaluate the dynamic flow properties of the material, which play a critical role in the application of PCMs for thermal management. The chemical crosslinking of the TTE epoxy chain ends with kraft lignin was confirmed by Fourier transform infrared spectroscopy (FTIR). FTIR measurements were performed within a spectrum from 400 to 4000 cm⁻^1^ and a resolution of 2 cm⁻^1^ using a Bruker Alpha II spectrometer (Bruker, Billerica, MA, USA) equipped with attenuated total reflection capability.

### 2.6. Experimental Setup

Electrical heaters were used in place of real batteries to ensure higher safety, provide a consistent and controllable heat source, and increase operability, enabling more precise measurements of the PCM’s thermal performance. The electrical heater was powered by a DC power supply, which provided 10 W, 15 W, and 17.5 W of power under different testing conditions. This power input generated heat in a controlled manner. A T-type thermocouple was attached directly to the surface of the heater to measure the temperature during operation. This thermocouple was connected to a data acquisition system, which recorded the temperature at a frequency of 10 measurements per second. This high sampling rate ensured continuous and precise monitoring of the heater’s temperature throughout the experiment. The recorded data were processed and visualized in real time using a connected PC, which displayed temperature graphs and stored the data for further analysis [30,31].

**Figure 2:**
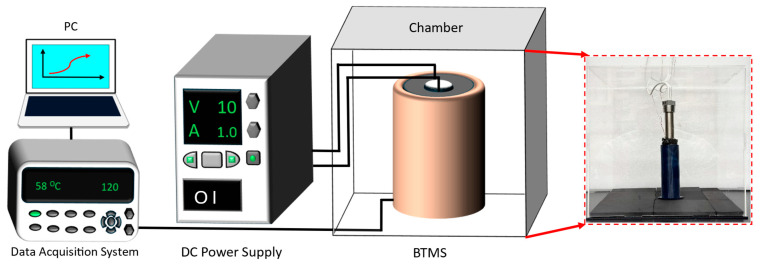
Schematic of the experimental setup used for evaluating the thermal performance of the battery thermal management system (BTMS) with lignin-modified PEO PCM. The setup includes a DC power supply, a data acquisition system, and a PC for real-time monitoring. The BTMS is housed in an acrylic box (shown in the photo on the right) to maintain consistent environmental conditions and minimize external thermal interference.

To minimize the influence of external environmental factors, the heater and PCM were enclosed in an acrylic box (Figure 2) [32]. The acrylic box isolated the system from ambient air disturbances, ensuring more accurate and reliable thermal measurements. The transparency of the acrylic material allowed for visual observation of the experiment while maintaining insulation. Each experiment began with the heater at ambient temperature to ensure consistent starting conditions [33,34]. The heater’s temperature rise was continuously monitored as power was supplied, and the thermal performance of the PCM was evaluated based on the time required to reach specific temperature thresholds, such as 60 °C. The entire assembly of the heater, PCM, and housing was integrated into a custom-designed BTMS, designed to simulate real-world battery thermal management conditions [35,36].

## 3. Results and Discussion

### 3.1. A Nature-Inspired Structure for PCM Development

**Figure 3:**
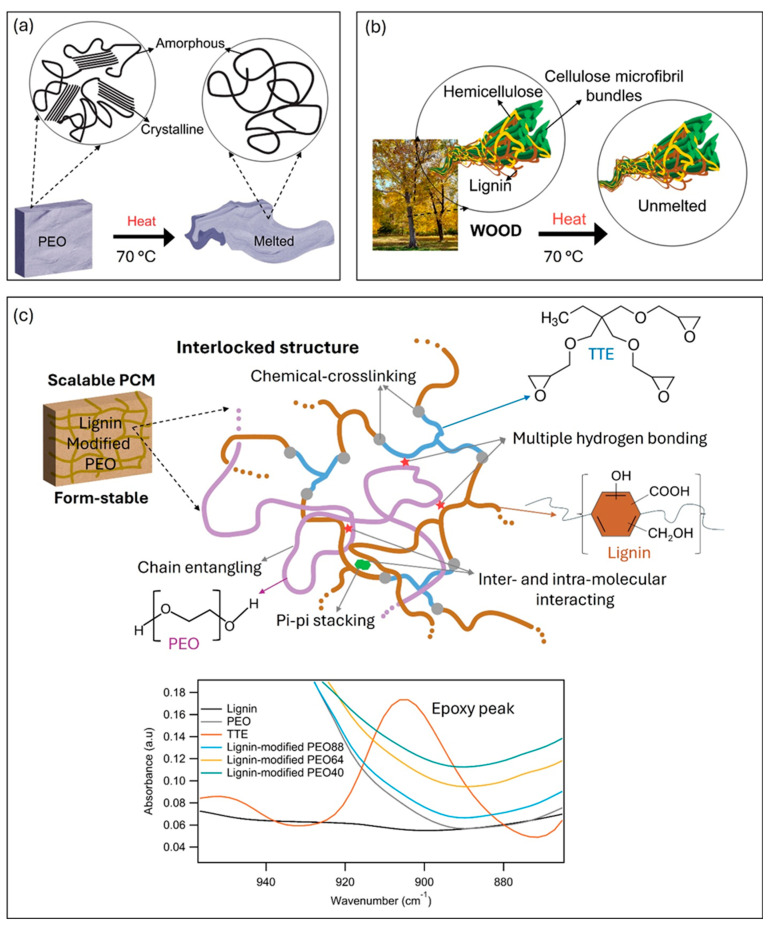
A nature-inspired structure of PCMs through interlocking kraft lignin with PEO: (**a**) The thermally unstable structure of PEO, (**b**) the thermally stable structure of wood when exposed to heat, and (**c**) lignin-modified PEO composites as scalable and form-stable PCMs through the formation of an interlocked structure. The presented FTIR spectra confirm the successful crosslinking between kraft lignin and TTE, as evidenced by the disappearance of the epoxy peak.

Nature-inspired materials are found in a wide range of applications [37,38]. Here, inspired by wood’s structure, we developed form-stable PCMs from lignin and PEO, as illustrated in Figure 3. PEO is a semicrystalline polymer with a low melting point of approximately 60 °C [39].

Due to its highly crystalline structure, PEO can absorb heat and undergo a phase transition, changing from solid to liquid as it transforms into an amorphous structure (Figure 3a). Its simple linear polymer chains facilitate a quick phase change at a temperature slightly above its melting point, with a low melt viscosity. Thus, a primary challenge in using PEO as a PCM is material leakage.

In contrast, wood is a natural composite with excellent thermal stability and does not melt [40]. Figure 3b shows the main components of a wood cell structure: cellulose, hemicellulose, and lignin. Cellulose exists in a crystalline form with strong hydrogen bonding, while lignin, with its highly branched, three-dimensional network, provides the primary support framework, giving wood its stiffness [41]. These strong molecular interactions, combined with lignin’s aromatic structure, yield high rigidity and thermal stability, preventing wood from melting at elevated temperatures. Our objective was to create a material that mimics wood’s structural stability while retaining PEO’s favorable melting behavior. To achieve this, we replicated wood’s natural cell structure by substituting cellulose with PEO and introducing kraft lignin and TTE crosslinkers to form a supporting framework for PEO (Figure 3c). By establishing an interlocked structure that includes multiple hydrogen bonds, π-π stacking, inter- and intramolecular interactions, chain entanglements within lignin and ultra-high molecular weight PEO, and covalent crosslinks between lignin and TTE epoxy chain ends, we successfully manipulated the phase change behavior of PEO chains. This structure exhibits excellent form stability, even when heated above 100 °C, well above PEO’s melting point.

The Fourier transform infrared spectroscopy (FTIR) data in Figure 3c demonstrate the disappearance of the epoxy peak in the lignin-modified PEO samples, indicating the reaction between the TTE epoxy chain ends and the hydroxyl and carboxyl groups of lignin. Full FTIR data for all samples are provided in Figure S1 (Supporting Information). The detailed molecular interactions and the interlocked structures formed between lignin, PEO, and the crosslinker were discussed in our prior work [24,25].

### 3.2. Thermal Characteristics of PCMs

The thermal stability of lignin-modified PEO composites contributes to their reworkability. After thermal cycling, when the material cools back down to room temperature, it solidifies without damage and can be easily detached from surfaces. This feature is particularly beneficial for applications like BTMS, where components often undergo maintenance or replacement [42]. The ability to detach and reapply the PCM without compromising its structural integrity enhances the material’s usability for long-term thermal management.

**Figure 4:**
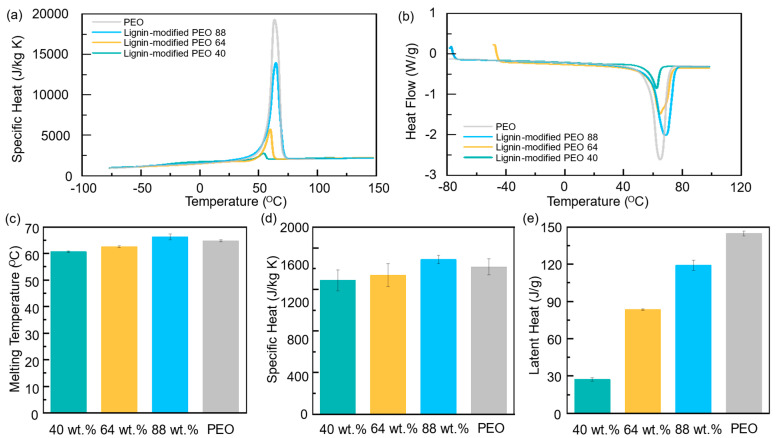
Thermal properties of lignin-modified PEO composites with varying PEO concentrations (40 wt.%, 64 wt.%, and 88 wt.%), including pure PEO for comparison. (**a**) Specific heat as a function of temperature, derived from DSC, indicating how each sample’s heat capacity changes near the melting temperature. (**b**) Heat flow as a function of temperature, derived from DSC. (**c**) Melting temperature, (**d**) specific heat capacity, and (**e**) latent heat values for each sample, highlighting the improved thermal stability and performance of lignin-modified composites compared to pure PEO. Standard deviation was obtained by repeating the measurements three times.

The thermal properties of the composites were tested across varying PEO concentrations (40 wt.%, 64 wt.%, and 88 wt.%), with pure PEO included as a control for comparison. As shown in Figure 4, the melting temperature, specific heat capacity, and latent heat increase with rising PEO concentrations. Both Figure 4a,b present data obtained from differential scanning calorimetry (DSC) measurements. Figure 4a illustrates the specific heat as a function of temperature, showing notable variations near the melting temperature, especially for higher PEO concentrations, highlighting the thermal response of each composite. Figure 4b shows the heat flow, where the peak areas correspond to the latent heat absorbed during phase transitions, reflecting the energy required for melting. Notably, pure PEO, while demonstrating similar specific heat and latent heat values, lacks the dimensional stability achieved in the lignin-modified PEO composites. The melting temperature of the composites rises from 60 °C at 40 wt.% PEO to nearly 70 °C at 88 wt.% PEO (Figure 4c), extending the operational range and enhancing suitability for high thermal loads. This stabilization effect can be directly attributed to lignin’s interlocking structure within the composites, preventing PCM leakage and maintaining a solid state even at elevated temperatures, a feature not observed in pure PEO. The higher PEO concentration also improves the specific heat capacity (Figure 4d), allowing the composite to absorb and store more thermal energy without a significant temperature rise. This property is critical for BTMS applications, where maintaining stable temperatures during charge–discharge cycles is essential for the longevity and safety of batteries [43].

Latent heat, which refers to the energy absorbed during phase transitions, also increases significantly with PEO concentration (Figure 4e). The composite with 88 wt.% PEO exhibits a latent heat of approximately 120 J/g, compared to only 20 J/g for the 40 wt.% PEO sample. This substantial increase in latent heat enables the PCM to manage larger thermal loads, effectively absorbing and releasing heat as needed without undergoing a complete phase change [44].

This combination of chemical and physical properties, PEO’s high heat capacity and thermal conductivity, combined with lignin’s structural stability and resistance to phase changes, makes the composite particularly suitable for applications like BTMSs. Unlike many conventional PCMs that degrade or become non-reworkable after repeated thermal cycles [45], the lignin-modified PEO composites remain stable, form-retentive, and reworkable. This durability, combined with its enhanced thermal management capabilities, positions the lignin-modified PEO composite as a strong alternative to commonly used PCMs in a variety of thermal management applications, especially those requiring sustainable, high-performance, and stable materials over extended use.

### 3.3. PCM Stability and Resistance to Melting

The stability of the PCM composites under elevated temperatures is one of its key advantages, particularly in comparison to conventional PCMs such as paraffin, which typically undergo a full phase transition into a liquid state [46,47]. This section examines the resistance of the composites to melting and their stability under thermal cycling, based on both mechanical and thermal characterizations.

**Figure 5:**
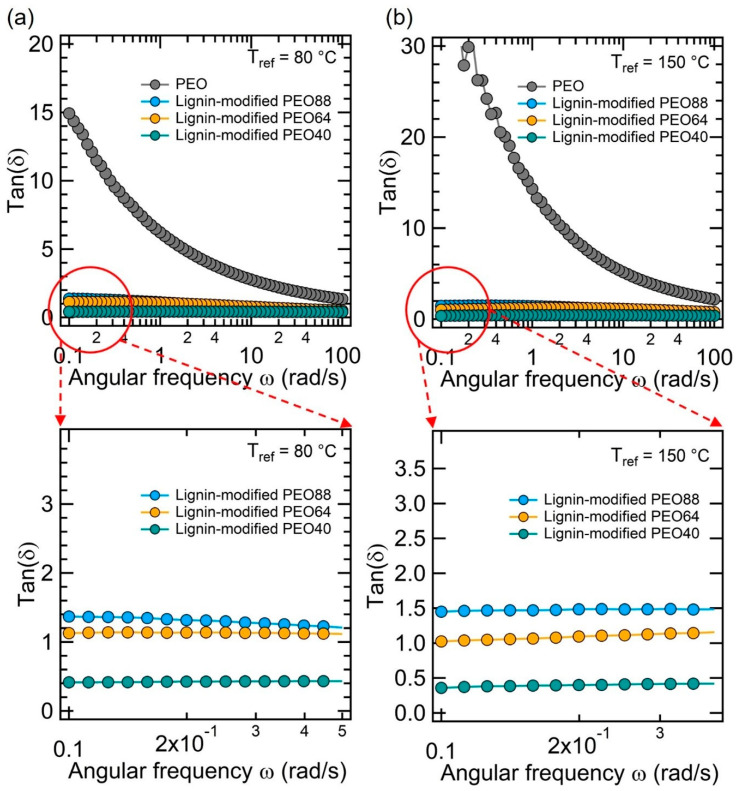
Loss tangent (tan(δ)) as a function of angular frequency for pure PEO and lignin-modified PEO composites (40 wt.%, 64 wt.%, and 88 wt.%) at (**a**) 80 °C and (**b**) 150 °C.

Figure 5 illustrates the mechanical stability of the lignin-modified PEO composites across different PEO concentrations (40 wt.%, 64 wt.%, and 88 wt.%) in both low (80 °C) and high (150 °C) temperature regimes. The two selected temperatures are far above their melting points, ca. 60–65 °C (Figure 4c). These measurements provide crucial insight into the viscoelastic behavior of the PCMs. As shown in Figure 5a,b, the loss tangent (tan(δ), a ratio of loss modulus over storage modulus) for the lignin-based PEO composites remains consistently low across a wide range of angular frequencies, indicating minimal viscous behavior, which corresponds to high material stability under heat. In all cases, the tan(δ) values of the lignin-modified PEO composites are nearly independent of the applied angular frequencies, which range from 0.1 to 100 rad/s. The zoomed-in graphs (marked with red circles) reveal low tan(δ) values for these samples, approximately between 0.5 and 1.5, at both reference temperatures of 80 °C and 150 °C. Notably, the sample containing 40 wt.% lignin shows a significantly low tan(δ) value of about 0.5. Additionally, the tan(δ) slightly increases with higher PEO content. Particularly at a higher temperature, 150 °C (Figure 5b), the lignin-modified PEO composites maintain their form-stable structure with little variation in tan(δ), even much above their melting points. In contrast, pure PEO shows a significant increase in tan(δ), which is indicative of a phase transition from solid to viscous behavior. The tan(δ) of PEO is highly influenced by the applied frequencies. For instance, at low frequencies and temperatures of 80 °C and 150 °C, its tan(δ) values are approximately 15 and 30, respectively. This suggests that the material exhibits a behavior similar to that of a complete liquid in its rest state.

As shown in Figure 4a–c, the higher PEO concentrations (40 wt.%, 64 wt.%, and 88 wt.%) provide increased latent heat and specific heat capacity, allowing for more efficient heat absorption and distribution. Despite reaching temperatures exceeding the melting point of pristine PEO (~65 °C), the lignin-modified PEO composites remain in a semi-solid state rather than transitioning to a liquid phase, as seen in Figure 5. This behavior is particularly important in applications where phase leakage or the loss of structural integrity can be detrimental to performance. This comparison highlights that the incorporation of lignin effectively prevents the lignin-modified PEO PCMs from entering a liquid state at elevated temperatures, thereby eliminating the risk of leakage.

**Figure 6:**
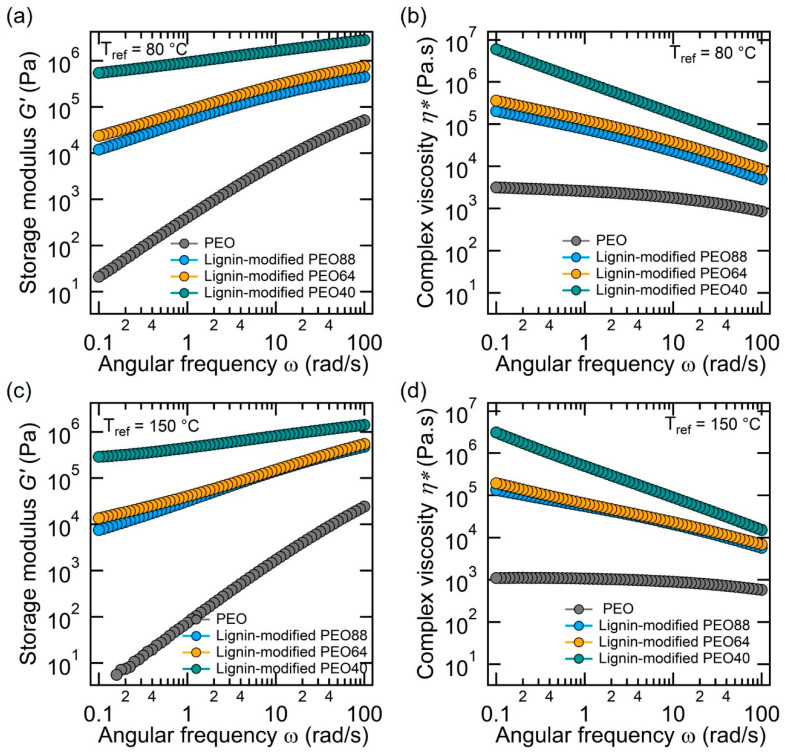
Storage modulus G′ (**a**,**c**) and complex viscosity η∗ (**b**,**d**) of pure PEO and lignin-modified PEO composites (40 wt.%, 64 wt.%, and 88 wt.% PEO) at reference temperatures of 80 °C (**a**,**b**) and 150 °C (**c**,**d**) across various angular frequencies.

Further evidence of the lignin-modified PEO PCMs’ stability can be observed in the storage modulus (G′) and complex viscosity (η*) [48], shown in Figure 6. These properties reveal that the composites maintain a high storage modulus across varying temperatures, with G′ values for the PEO composites remaining significantly higher than those of pristine PEO in both reference temperatures. For instance, at a temperature of 150 °C and a frequency of 0.1 rad/s, the storage modulus and complex viscosity of the 88 wt.% PEO composite (represented by the blue curves in Figure 6c,d) are more than three and two orders of magnitude higher than those of pristine PEO, respectively. This indicates that the material can maintain its elasticity and structural integrity even under high thermal loads. The high values of the complex viscosity of the lignin-modified PEO composites indicate their capacity to resist flow and maintain stable thermal performance, which is critical for long-term applications such as BTMS.

The resistance to melting is attributed to the unique interlocked structure formed through chemical and physical crosslinks within PEO and lignin as shown in Figure 3c. This structure prevents the lignin-modified PEO composites from undergoing a full-phase transition from solid to liquid at temperatures above the melting point of PEO, resulting in high dimensional stability for the composites. Even at temperatures as high as 150 °C, the lignin-modified PEO composites show significant improvements in complex viscosity and storage modulus compared to pristine PEO. This indicates a remarkable resistance to melting and deformation, making these composites suitable for a wider range of applications in high-temperature and high-stress conditions.

Moreover, the design of an interlocking structure within lignin and PEO improves the material’s reworkability. After thermal cycling, the composite can be easily detached from surfaces once cooled to room temperature without any significant degradation in performance. This is a notable improvement over conventional PCMs, which often undergo phase changes and cannot be reused without compromising their structural integrity. The form-stable nature of the lignin-modified PEO composites, combined with their excellent resistance to melting and deformation, ensures consistent performance over multiple thermal cycles, making these composites highly suitable for long-term thermal management applications. In our study, we demonstrated the stability of the lignin-modified PEO composites under varying thermal conditions. These results will be discussed in the following sections. The obtained data suggest their potential for use in applications that demand not only structural integrity but also consistent thermal performance over extended periods. The composites’ resistance to phase transitions and their ability to manage significant thermal loads position them as a robust solution in high-stress environments.

To further understand how this stability impacts thermal response in practical applications, the next section delves into a detailed temperature analysis, examining how varying PEO concentrations influence heat absorption and temperature regulation under different power inputs.

### 3.4. Detailed Thermal Management Analysis

The detailed thermal management experiment was designed to investigate the thermal response of the lignin-modified PEO composites at varying PEO concentrations (40 wt.%, 64 wt.%, and 88 wt.%) under different power settings. As discussed in the previous sections, pristine PEO lacks form stability, readily transitions to a liquid phase, and cannot be reprocessed through multiple thermal cycles. Consequently, PEO was excluded from this experiment. The objective of this analysis was to observe how PEO concentration affects the time it takes for the heater to reach key temperature thresholds, specifically 60 °C and 110 °C, when exposed to power inputs of 10 W, 15 W, and 17.5 W. The two temperatures were selected based on the melting point of PEO [49], one was close to it, while the other was significantly higher but still within the heater’s capacity.

**Figure 7:**
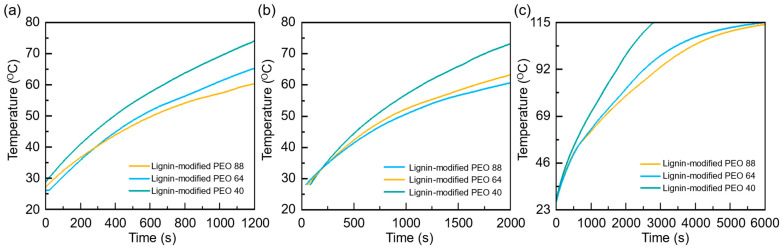
Temperature profiles of lignin-modified PEO composites with varying PEO concentrations (40 wt.%, 64 wt.%, and 88 wt.%) under power inputs of (**a**) 10 W, (**b**) 15 W, and (**c**) 17.5 W. The graphs illustrate the temperature rise over time, highlighting the influence of PEO concentration on thermal response and heat absorption capabilities.

At 10 W, the temperature profiles (Figure 7a) reveal a clear trend: as the PEO concentration increases, the time taken for the composite to reach 60 °C significantly extends. The sample with 40 wt.% PEO reached 60 °C in 1153 s, the 64 wt.% PEO sample took 1662 s, and the 88 wt.% PEO sample reached 60 °C after 1910 s. This highlights the significant delay in temperature rise as PEO concentration increases, which can be attributed to its enhanced heat absorption capacity due to higher latent heat and specific heat values. As demonstrated in Figure 4b,c, the 88 wt.% PEO composite exhibits considerably higher specific heat and latent heat compared to the lower-concentration samples. Specifically, the latent heat and specific heat of the 88 wt.% PEO, 64 wt.% PEO, and 40 wt.% PEO composites are 119 J/g and 1858 J/kg K, 92 J/g and 1774 J/kg K, and 27 J/g and 1608 J/kg K, respectively. Therefore, this allows the 88 wt.% PEO composite to store more thermal energy without a rapid temperature rise. This capacity to absorb and store large amounts of heat is vital in thermal management systems, where a delayed temperature increase is often desirable for preventing overheating.

The same pattern is observed in the 15 W experiment (Figure 7b), although the overall times were reduced due to the higher power input. For instance, the 40 wt.% PEO reached 60 °C in 676 s, the 64 wt.% PEO took 954 s, and the 88 wt.% PEO sample took 1171 s. Nevertheless, the relative impact of the PEO concentration remained consistent, with the 88 wt.% PEO composite once again taking the longest to reach 60 °C. This delay in heating further demonstrates the composite’s efficiency in managing thermal loads, as its higher latent heat allows it to buffer against sudden temperature increases, maintaining stability over a longer period.

When the power input was increased to 17.5 W, as shown in Figure 7c, the heater was pushed to a higher temperature range, extending the analysis to 110 °C. At this higher power setting, the 88 wt.% PEO composite continued to exhibit a slower temperature rise compared to the other compositions. The 88 wt.% PEO sample reached 110 °C after 4846 s, significantly longer than the 64 wt.% PEO which took 4390 s and 40 wt.% PEO also took 2499 s. The extended time required to reach 110 °C can be directly linked to the substantial increase in latent heat capacity with rising PEO concentrations (Figure 4e). The 88 wt.% PEO composite exhibited a latent heat of 119 J/g, significantly higher than the 40 wt.% PEO sample, which only exhibited 27 J/g. This higher latent heat allows the composite to manage larger thermal loads effectively, absorbing heat without undergoing a full-phase change, which is essential for maintaining stable performance under high thermal stress.

Pure PEO was considered as a control sample; however, its rapid deformation under heat and inability to maintain structural integrity make it unsuitable for practical applications. This behavior limits its utility as a reliable PCM for thermal management systems, especially under high thermal cycling conditions. Figure S3 (Supporting Information) includes photographic evidence of pure PEO’s severe thermal deformation, further illustrating its limitations.

Therefore, a comparative analysis was conducted using previously reported data on paraffin wax, a commonly used PCM in thermal management applications. In the study by Zheng et al. [31], paraffin wax was tested in a cylindrical BTMS system with experimental conditions similar to those in this study. Specifically, the heater configuration and thermocouple placement closely resemble those used for the lignin-modified PEO composites, enabling a meaningful comparison of thermal performance.

For paraffin wax, the times required to reach 60 °C under varying power inputs were reported as follows: 1060 s at 10 W, 810 s at 12.5 W, and 620 s at 15 W [31]. In comparison, the lignin-modified PEO composites exhibited significantly improved performance. At 10 W, the 88 wt.% PEO composite required 1910 s to reach 60 °C, nearly doubling the time of paraffin wax under identical conditions. Similarly, at 15 W, the 88 wt.% PEO composite took 1171 s to reach 60 °C, demonstrating its superior heat absorption and delayed temperature rise capabilities.

While the paraffin wax data do not extend to the higher power input of 17.5 W, the performance of the 88 wt.% PEO composite under this condition further highlights its thermal efficiency. Specifically, it required 4846 s to reach 110 °C, demonstrating its ability to manage larger thermal loads while maintaining stability. This capacity is particularly crucial for high-stress applications such as BTMSs, where a slower temperature rise can prevent overheating and ensure operational safety.

This comparison demonstrates that the lignin-modified PEO composite offers a more reliable and effective solution for thermal management applications compared to conventional PCMs such as paraffin wax. By combining superior thermal properties with enhanced structural integrity, the composite represents a significant step forward in PCM technology for high-demand environments. These superior thermal properties are further complemented by the composite’s remarkable structural stability, which is explored in the following discussion.

In addition to the slower temperature rise, it was observed that the lignin-modified PEO composites remained solid even when exposed to temperatures exceeding 110 °C. While many conventional PCMs, including pristine PEO, undergo a phase transition to liquid when exposed to high temperatures, the lignin-modified PEO composites maintained their solid-state structure throughout the experiment. This structural stability is primarily due to the lignin’s reinforcement within the interlocking structure of the composites, which prevents the material from transitioning to a liquid phase, even under extreme heat conditions. The solid-state behavior at elevated temperatures ensures that the PCMs do not experience leakage, a common issue in conventional PCMs like paraffin wax. This makes the lignin-modified PEO composites particularly suitable for high-temperature applications such as BTMSs, where long-term stability and form retention are critical.

The specific heat of the composite also plays a critical role in the observed temperature response [50]. As shown in Figure 4d, the specific heat capacity of the lignin-modified PEO composite increases with higher PEO concentrations. This increase enables the composite to absorb more heat over time, delaying the overall temperature rise. The higher specific heat and latent heat observed in the 88 wt.% PEO composite are key factors in its ability to delay the heater’s temperature increase under high-power conditions. These thermal properties provide a significant advantage in thermal management applications, where managing heat over prolonged cycles is vital for ensuring the system’s safety and longevity.

### 3.5. Thermal Adhesion and Enhanced Heat Transfer

One of the standout features of the lignin-modified PEO PCMs is its ability to maintain thermal adhesion and significantly enhance heat transfer, particularly in demanding applications such as BTMSs. When exposed to elevated temperatures, the composites’ unique structure allows them to form a tight thermal interface with heat sources, ensuring consistent and efficient heat distribution. This section delves into the composites’ behavior under high-temperature conditions and explores how their thermal properties contribute to improved heat management performance.

**Figure 8:**
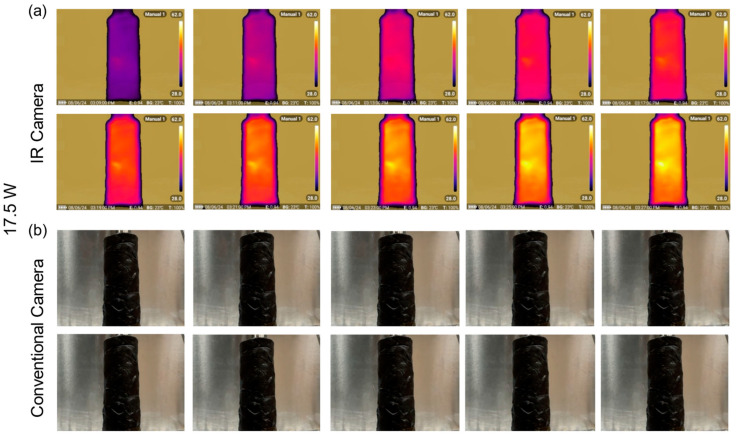
Thermal distribution images of the lignin-modified PEO composite PCM with 64 wt.% PEO concentration at 17.5 W power input. (**a**) Infrared (IR) camera images showing uniform heat distribution across the PCM, indicating strong thermal adhesion and effective heat transfer between the PCM and heater. (**b**) Conventional camera images illustrating the material’s dimensional stability without significant deformation or sagging under prolonged heating.

As demonstrated in Figure 8a, the IR camera images show examples of the lignin-modified PEO composite with 64 wt.% PEO concentration, exhibiting an even heat distribution when subjected to a power input of 17.5 W. The heat spreads uniformly across the surface of the heater and the PCM, highlighting the strong thermal interface formed between the composite and the heater. This uniform heat distribution is essential in thermal management applications [51], as it prevents localized overheating and ensures that the PCM can absorb and transfer heat effectively. The thermal distribution images for PEO concentrations of 40 wt.% and 88 wt.% are available in the Supporting Information, providing further insights into the temperature profile consistency across different composite formulations under similar conditions.

The enhanced thermal adhesion of the lignin-modified PEO composites can be attributed to their balanced viscoelastic behavior, which allows the material to form a tight contact with the heater surface without undergoing significant flow or deformation. As the PCMs soften slightly at elevated temperatures, they fill the gaps between the heater and the composite, improving interfacial contact and heat transfer, and reducing the thermal resistance at the interface. This surface compliance, without excessive flow, ensures that the materials maintain close contact with the heat source, optimizing heat conduction from the heater to the PCMs.

The conventional camera images (Figure 8b) provide further evidence of the composite’s structural stability under high temperatures. Unlike conventional PCMs that may undergo phase changes and deform, this lignin-modified PEO PCM retains its form even when exposed to prolonged heating. This dimensional stability is crucial in long-term applications where the material must maintain its structural integrity while still transferring heat efficiently. The PCM does not sag or lose its shape, ensuring consistent thermal performance over extended periods.

Moreover, the addition of lignin to the PEO matrix enhances this balance between rigidity and surface compliance. Pure PEO, when subjected to high temperatures, tends to become overly viscous and liquid, leading to uneven adhesion and the formation of hotspots on the heater surface. However, the introduction of lignin and the interlocked structure moderates this behavior, allowing the composite to soften just enough to conform to the surface without losing its structural integrity. This results in a smoother and more consistent heat transfer across the entire surface of the PCM, as shown in the IR camera images.

This controlled surface softening observed in the lignin-modified PEO PCMs enhances its ability to act as a thermal interface material (TIM), facilitating efficient heat transfer from the heater to the surrounding environment. This behavior is particularly valuable in BTMSs, where maintaining stable temperatures across the battery pack is critical for preventing thermal runaway and prolonging battery life [52]. The close adhesion between the PCMs and the heater surface ensures that heat is evenly distributed, minimizing the formation of thermal hotspots and ensuring uniform temperature control.

In comparison to conventional PCMs, which often exhibit excessive flow and phase leakage at high temperatures, the lignin-modified PEO composites offer a more controlled and stable response. Their capability to maintain contact with the heater while avoiding excessive deformation, melting, and liquid flow makes them an ideal choice for high-temperature applications where consistent thermal regulation is essential.

By combining thermal adhesion with dimensional stability, the lignin-modified PEO PCMs demonstrated a unique capability to enhance heat transfer while maintaining long-term performance. The materials maintain form stability while still adapting to the heater surfaces, ensuring efficient contact and thermal management, making them ideal for high thermal loads and long-term operational stability.

## 4. Conclusions

This study presents a strategy in design of lignin-based PEO PCMs through the formation of an interlocked structure using both chemical and physical crosslinking of lignin–PEO composites via co-reactive melt processing. The detailed thermal management analysis of developed lignin-modified PEO composites exhibits promising results that can address critical limitations of conventional PCMs, including material leakage, dimensional instability, and environmental concerns. By leveraging PEO’s high heat capacity and thermal conductivity alongside lignin’s inherent rigidity and flame resistance, the composite demonstrates enhanced form stability, reworkability, and effective thermal adhesion under elevated temperatures and repeated thermal cycling. The interlocked structure not only prevents phase transition from solid to liquid but also maintains the materials’ form-stable performance, making them ideal for applications in BTMSs, TIMs, and other high-demand environments such as electric vehicle power systems and high-performance computing system cooling. Altogether, these unique properties position the lignin-modified PEO composites as robust, environmentally compatible alternatives, offering reliable, long-lasting thermal regulation and significantly advancing PCM technology for sustainable thermal management solutions.

## Data Availability

Data in the paper and supporting information are available from the corresponding authors upon request.

## Supplementary Materials

The following supporting information can be downloaded at: https://www.mdpi.com/article/10.3390/polym17010044/s1, Figure S1. Full Fourier transform infrared (FTIR) data for lignin, PEO, TTE, and lignin-modified PEO composites with varying PEO concentrations (40 wt.%, 64 wt.%, and 88 wt.%). Figure S2. Thermal distribution images of the lignin-modified PEO composite PCM with a 40 wt.% PEO concentration at a 17.5 W power input. (**a**) Infrared (IR) camera images. (**b**) Conventional camera images. Figure S3. Thermal distribution images of the lignin-modified PEO composite PCM with a 88 wt.% PEO concentration at a 17.5 W power input. (**a**) Infrared (IR) camera images. (**b**) Conventional camera images. Figure S4. Images of pure PEO sample before and after heat exposure. The “Before” image shows the initial structural integrity of the pure PEO sample, while the “After” image illustrates its deformation following thermal exposure. This demonstrates pure PEO’s susceptibility to losing its form under elevated temperatures, supporting the necessity of structural enhancement through lignin modification, as discussed in the main text. Figure S5. Differential scanning calorimetry (DSC) curves of pure PEO and lignin-modified PEO composites (40 wt.%, 64 wt.%, and 88 wt.%) from the first additional set of experiments conducted to validate the specific heat and heat flow characteristics as functions of temperature. Figure S6. Differential scanning calorimetry (DSC) curves of pure PEO and lignin-modified PEO composites (40 wt.%, 64 wt.%, and 88 wt.%) from the second additional set of experiments conducted to further confirm and complement the previous measurements.
