# Supplementary material for: Lignin–Polyethylene Oxide Interlocked Phase Change Materials with Enhanced Thermal Stability and Form Retention for Efficient Heat Management
Source: Polymers (Basel). 2024 Dec 28;17(1):44. doi: 10.3390/polym17010044 (PMC11722640; doi:10.3390/polym17010044)
Supplement: Supplementary file 1 [file polymers-17-00044-s001.zip › polymers-3346264-supplementary.pdf]

Supplementary Materials

Lignin–Polyethylene Oxide Interlocked Phase Change Materials with Enhanced Thermal Stability and Form Retention for Efficient Heat Management

JunSang Park [1], Pranto Karua [1], Songtao Tang [1], Ngoc A. Nguyen [2,*] and Lili Cai [1,3,*]

[1] Department of Mechanical Science and Engineering, The Grainger College of Engineering, University of Illinois Urbana-Champaign, Urbana, IL 61801, USA; junsang5@illinois.edu (J.S.P.); pkarua2@illinois.edu (P.K.); songtat@illinois.edu (S.T.)
[2] Illinois Applied Research Institute, The Grainger College of Engineering, University of Illinois Urbana-Champaign, Champaign, IL 61801, USA
[3] Materials Research Laboratory, The Grainger College of Engineering, University of Illinois Urbana-Champaign, Urbana, IL 61801, USA
* Correspondence: nanguyen@illinois.edu (N.A.N.); lilicai@illinois.edu (L.C.)

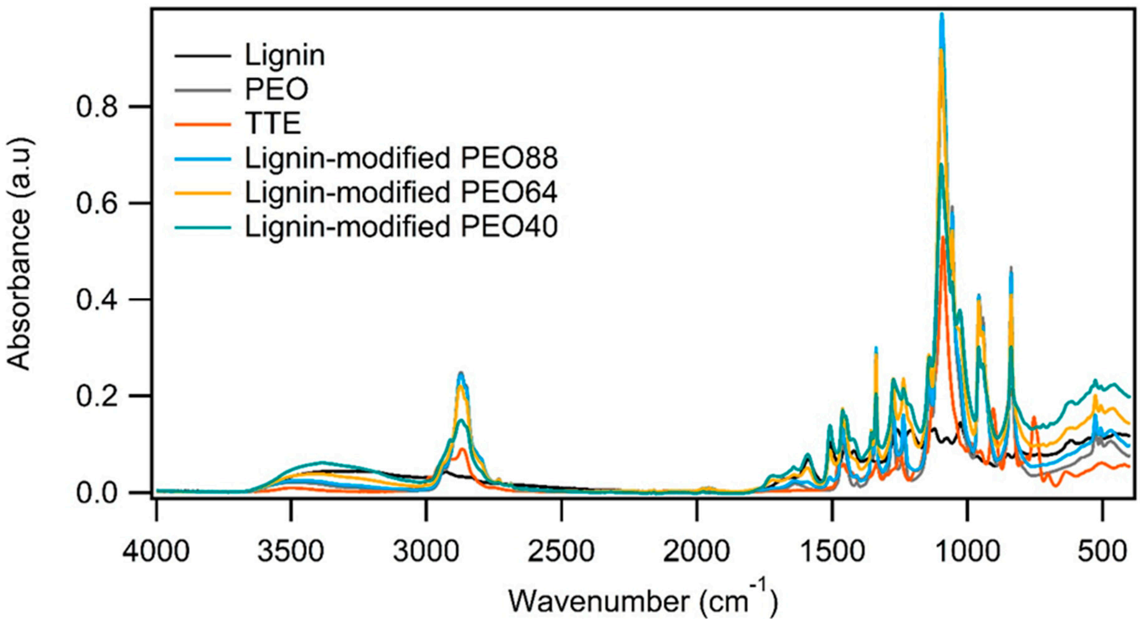

Figure S1. Full Fourier-transform infrared (FTIR) data of lignin, PEO, TTE, and lignin-modified PEO composites with varying PEO concentrations (40 wt.%, 64 wt.%, and 88 wt.%).

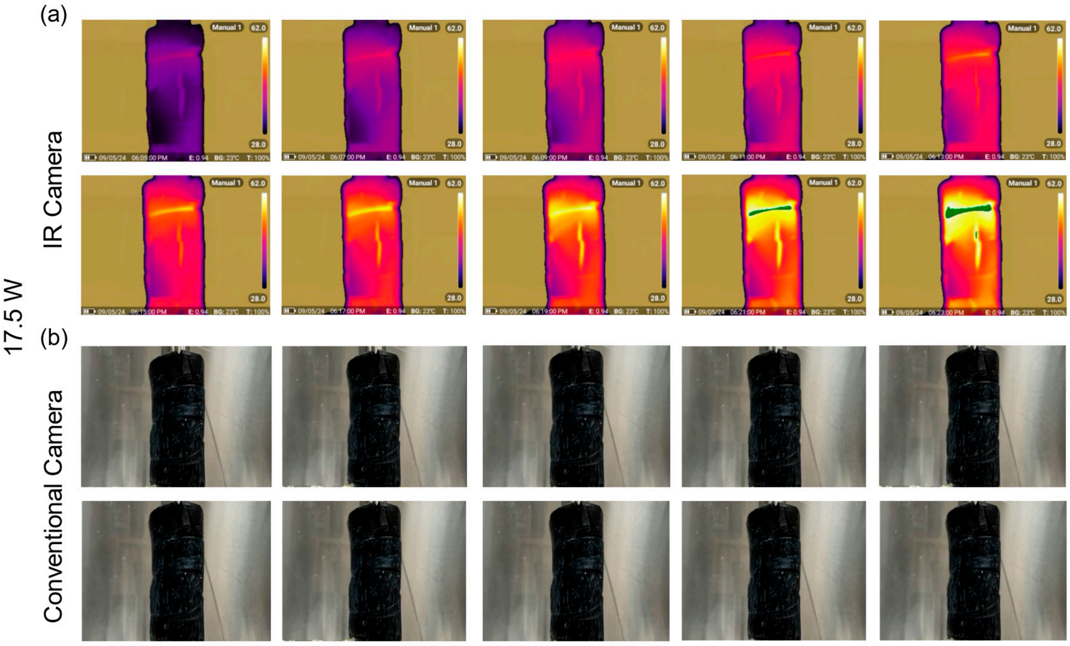

Figure S2. Thermal distribution images of the lignin-modified PEO composite PCM with 40 wt.% PEO concentration at 17.5 W power input. (a) Infrared (IR) camera images. (b) Conventional camera images.

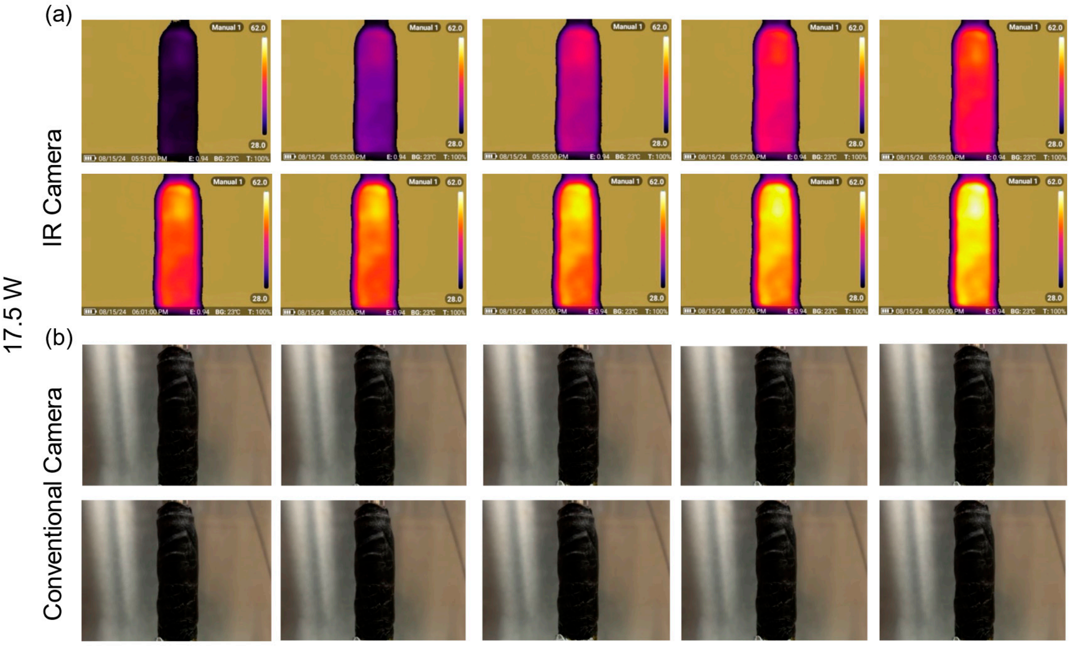

Figure S3. Thermal distribution images of the lignin-modified PEO composite PCM with 88 wt.% PEO concentration at 17.5 W power input. (a) Infrared (IR) camera images. (b) Conventional camera images.

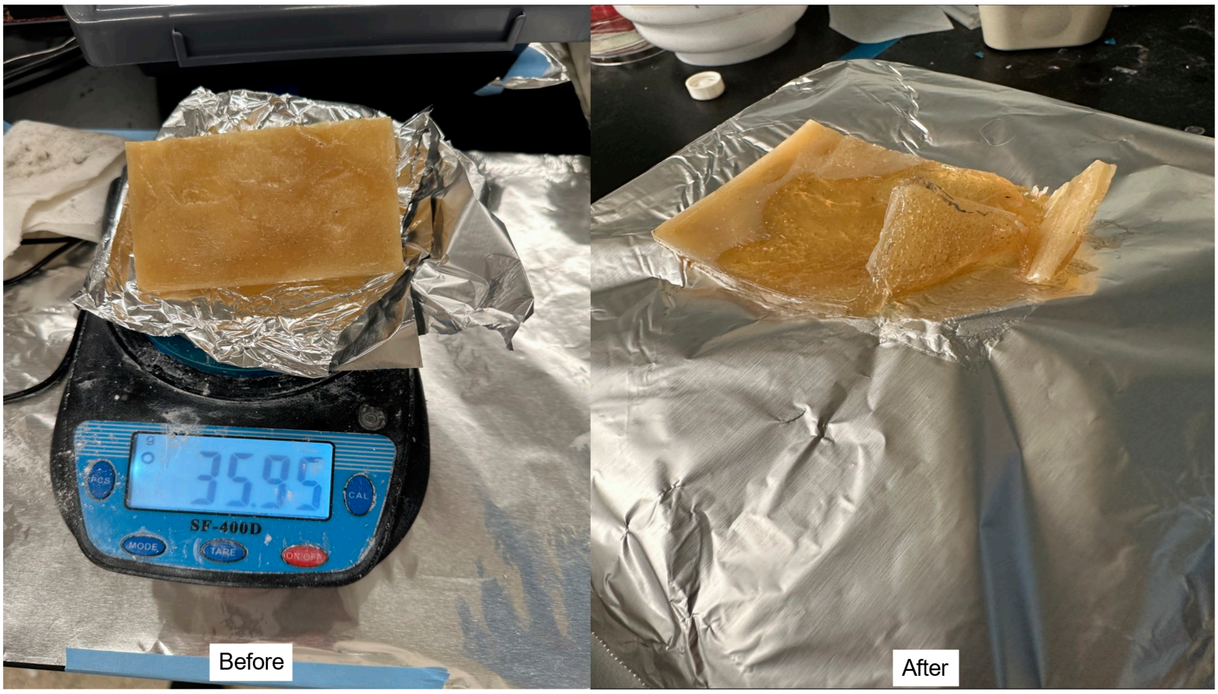

Figure S4. Images of pure PEO sample before and after heat exposure. The “Before” image shows the initial structural integrity of the pure PEO sample, while the “After” image illustrates its deformation following thermal exposure. This demonstrates pure PEO's susceptibility to losing its form under elevated temperatures, supporting the necessity of structural enhancement through lignin modification, as discussed in the main text.

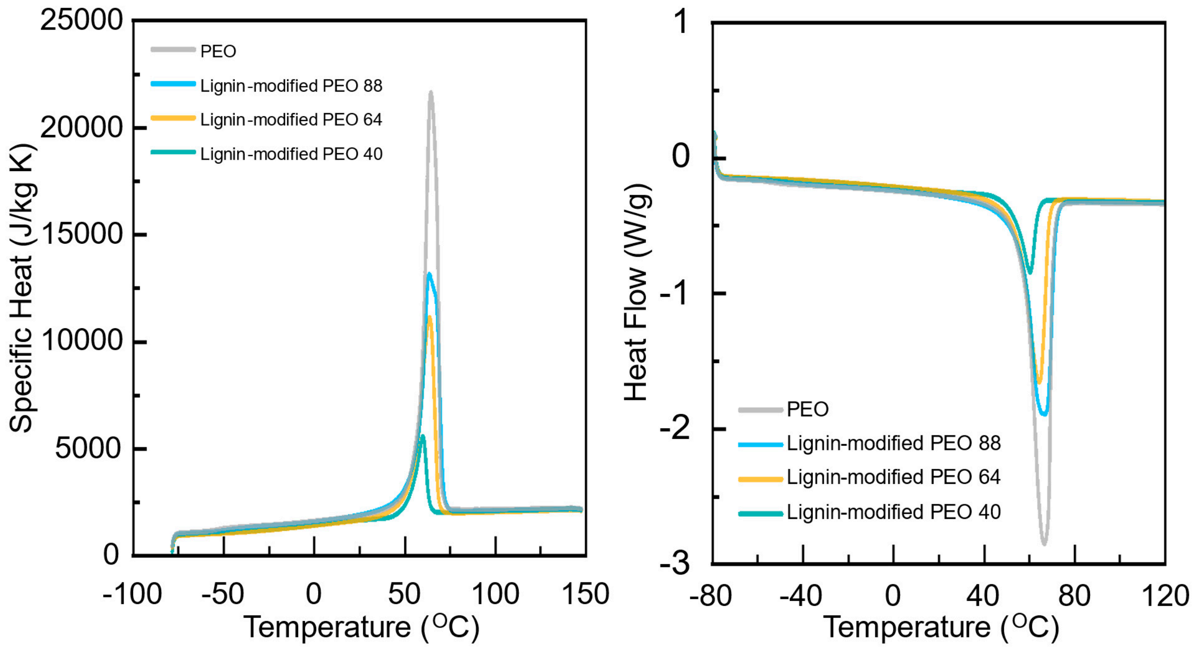

Figure S5. Differential Scanning Calorimetry (DSC) curves of pure PEO and lignin-modified PEO composites (40 wt.%, 64 wt.%, and 88 wt.%) from the first additional set of experiments conducted to validate the specific heat and heat flow characteristics as functions of temperature.

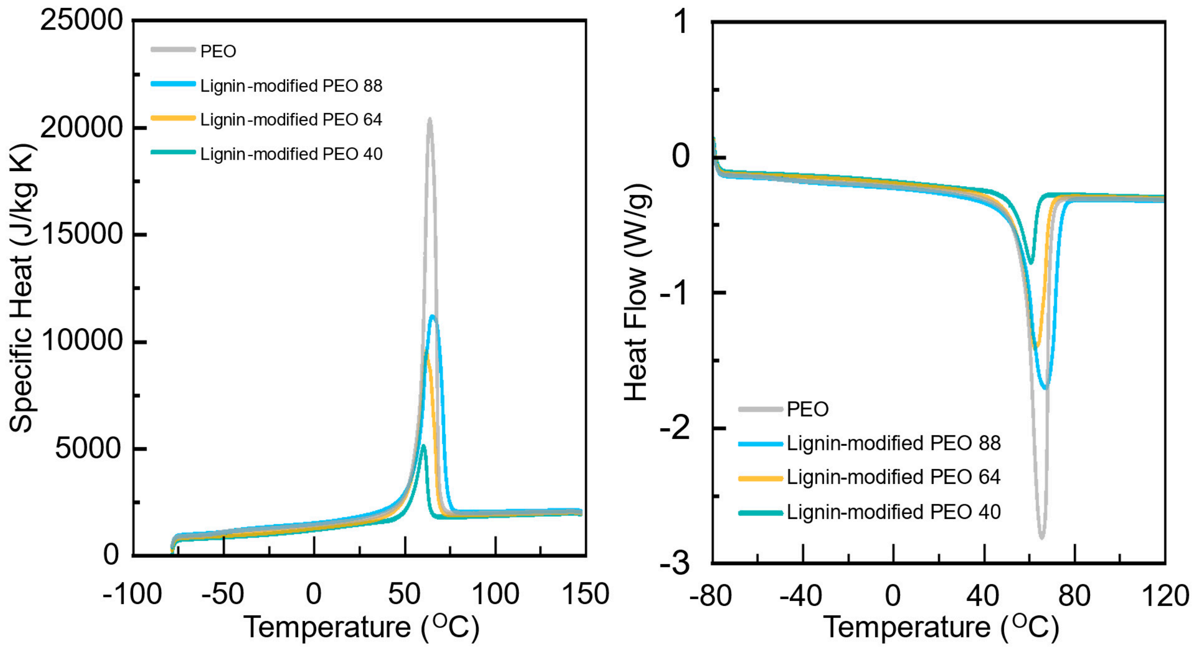

Figure S6. Differential Scanning Calorimetry (DSC) curves of pure PEO and lignin-modified PEO composites (40 wt.%, 64 wt.%, and 88 wt.%) from the second additional set of experiments conducted to further confirm and complement the previous measurements.
